# Prevalence of adenomyosis in endometrial cancer patients: a systematic review and meta-analysis

**DOI:** 10.1007/s00404-020-05840-8

**Published:** 2020-10-23

**Authors:** Antonio Raffone, Renato Seracchioli, Diego Raimondo, Manuela Maletta, Antonio Travaglino, Ivano Raimondo, Ilaria Giaquinto, Benedetta Orsini, Luigi Insabato, Massimiliano Pellicano, Fulvio Zullo

**Affiliations:** 1 Gynecology and Obstetrics Unit, Department of Neuroscience, Reproductive Sciences and Dentistry, School of Medicine, University of Naples Federico II, Naples, Italy; 2 Gynecology and Human Reproduction Physiopathology, Dipartimento Di Scienze Mediche E Chirurgiche (DIMEC), IRCCS S. Orsola Hospital, University of Bologna, Bologna, Italy; 3 Pathology Unit, Department of Advanced Biomedical Sciences, School of Medicine, University of Naples Federico II, Naples, Italy; 4 Gynecologic and Obstetric Unit, Department of Medical, Surgical and Experimental Sciences, University of Sassari, Sassari, Italy; 5 School in Biomedical Sciences, University of Sassari, Sassari, Italy

**Keywords:** Endometrium, Myometrium, Tumor, Malignancy, Gynecology, Oncology

## Abstract

**Introduction:**

Several studies have assessed the histological co-existence of endometrial carcinoma (EC) and adenomyosis. However, the significance of this association is still unclear.

**Objective:**

To assess the prevalence of adenomyosis in women with EC for a better understanding of the association between the two diseases.

**Materials and methods:**

A systematic review and meta-analysis was performed by searching electronics databases from their inception to March 2020, for all studies that allowed extraction of data about prevalence of adenomyosis in EC patients. Adenomyosis prevalence was calculated for each included study and as pooled estimate, with 95% confidence interval (CI).

**Results:**

Eight retrospective cohort studies assessing 5573 EC patients were included in our analysis. Of total, 1322 were patients with adenomyosis, and 4251 were patients without adenomyosis. Pooled prevalence of adenomyosis in EC patients was 22.6% (95% CI 12.7–37.1%).

**Conclusion:**

Adenomyosis prevalence in EC patients was not different from that reported for other gynecological conditions. The supposed association between the two diseases appears unsupported.

## Introduction

Adenomyosis is a benign gynecologic condition, defined as the migration of glands and stroma from basal layer of the endometrium to the myometrium [1]. Exposure to estrogens, parity and prior uterine surgery are considered as risk factors for the disease [1]. The most popular theory is that the alteration or absence of the endometrial–myometrial interface (junctional zone) may promote the pathological invagination of the endometrial mucosa into the muscle fibers of the myometrium [2]. Heavy menstrual bleeding and dysmenorrhea are main clinical manifestations, while infertility, dyspareunia and chronic pelvic pain are less common. Nevertheless, one-third of patients are asymptomatic [1–5].

Several studies have assessed the histological co-existence of endometrial carcinoma (EC) and adenomyosis reporting different data [6–13]. The two pathologies share common etiopathogenetic mechanisms including unopposed hyper-oestrogenic state, inflammatory milieu and molecular features favoring cell proliferation and inflammation [14–20]. According to some authors, adenomyosis could promote EC and affect pathological factors impacting on EC prognosis [7, 8, 11]. In fact, EC with adenomyosis has been associated with better overall survival, early International Federation of Gynaecology and Obstetrics (FIGO) stage and low FIGO grade [7, 8]. However, findings by studies assessing the association between adenomyosis and EC were affected by a low sample size, and this association is still unclear to date.

The objective of this systematic review and meta-analysis was to assess the prevalence of adenomyosis in women with EC to provide a deeper understanding of the association between the two diseases.

## Materials and methods

### Study protocol

Each review step was performed following an a priori designed study protocol, and the whole study was reported following the Preferred Reporting Item for Systematic Reviews and Meta-analyses (PRISMA) statement and checklist [21]. Each review step was independently performed by two authors, and disagreements were solved by discussion with all authors.

### Search strategy and study selection

Seven electronic databases (i.e., Web of Sciences, Google Scholar, Scopus, MEDLINE, ClinicalTrial.gov, Cochrane Library, and EMBASE) were searched from their inception to March 2020 using the following text words in different combinations: “endometr*”; “malignancy”; “tumour”; “tumor”; “neoplas*”; “cancer”; “carcinoma”;.”adenomyosis”; “myometr*”. References list from each eligible study was also screened for searching any studies missed during the electronic databases search.

All peer-reviewed studies that allowed extraction of data about prevalence of adenomyosis in EC patients were included in this study. A priori defined exclusion criteria were: case reports, literature review, studies with patient selection based on EC population characteristics (and thus affecting adenomyosis prevalence).

### Risk of bias within studies assessment

The risk of bias within studies assessment was performed by following The Methodological Index for Non-Randomized Studies (MINORS) [22]. Six domains related to risk of bias were applicable and were assessed for each included study: (1) Aim (had the study a clearly stated aim?); (2) Patient selection (were all eligible patients included during the study period?); (3) Data collection (were data collection performed following a protocol a priori defined?); (4) Endpoints (were outcomes clearly stated?); (5) Endpoint assessment (were histological diagnostic criteria for adenomyosis clearly stated?); (6) Loss to follow-up less than 5% (were patients with no information about adenomyosis diagnoses less than 5% of total sample?).

Authors judged each included study for each domain as “low risk”, “unclear risk”, or “high risk” if data were “reported and adequate”, “not reported” or “reported but inadequate”, respectively.

### Risk of bias across studies assessment

The risk of bias across studies assessment was performed by a funnel plot that reported the included studies on a plan based on logit adenomyosis rate in endometrial cancer patients (on the *x* axis) and the standard error (on the *y* axis). The risk of publication bias was considered significant if the funnel plot was asymmetrical and/or if the studies with higher standard error (which indicate low study accuracy) showed higher adenomyosis prevalence.

### Data extraction and analysis

Data from the included studies were extracted without modification according to the PICO (Population, Intervention, Comparator, Outcomes) items [23–26].

“Population” of our study was patients with EC.

“Intervention” (or risk factor) was the diagnosis of adenomyosis.

“Comparator” was not applicable since the study was designed as systematic review and meta-analysis of prevalence.

“Outcome” was the prevalence of adenomyosis in patients with EC.

Prevalence of adenomyosis in patients with EC was calculated as the number of patients with adenomyosis by the total number of patients with EC. Prevalence was calculated for each included study and as pooled estimate, and shown on forest plots with 95% confidence interval (CI). The random effect model of DerSimonian and Laird was used for all analyses.

Statistical heterogeneity among the included studies was evaluated by the inconsistency index *I*^2^, and judged as: null for *I*^2^ = 0%, minimal for *I*^2^ < 25%, low for *I*^2^ < 50%, moderate for *I*^2^ < 75% and high for *I*^2^ ≥ 75%, as previously described [24].

Data analysis was performed using Review Manager 5.3 (Copenhagen: The Nordic Cochrane Centre, Cochrane Collaboration, 2014) and Comprehensive Meta-Analysis (Biostat, 14 North Dean Street, Englewood, NJ 07631, USA) as software.

## Results

**Fig. 1:**
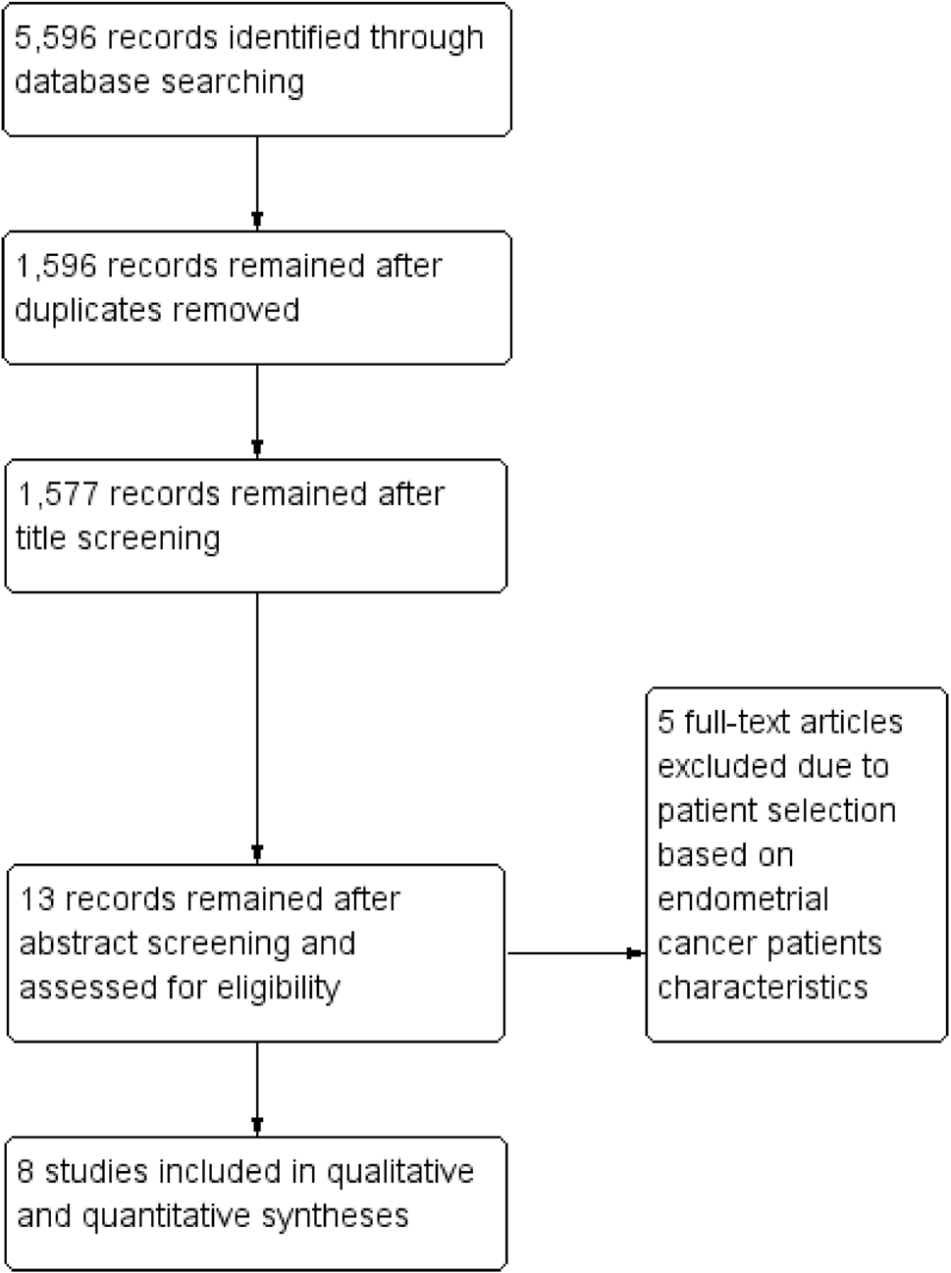
Flow diagram of studies identified in the systematic review [Prisma template (Preferred Reporting Item for Systematic Reviews and Meta-analyses)]

Electronic searches identified 5596 studies. 1596 studies remained after duplicates removal. 1577 studies remained after title screening. 13 articles remained after abstracts screening, and were assessed for eligibility. Finally, eight studies were included in the qualitative and quantitative analyses [6–13] (Fig. 1).

All the included studies were designed as retrospective cohort studies, and assessed a total of 5573 patients with EC (Table 1). Of total, 1322 were patients with adenomyosis, and 4251 were patients without adenomyosis (Table 2). Mean age of patients ranged from 50.7 to 64.2 years, and mean Body Mass Index ranged from 25.2 to 35.8 kg/m^2^.

**Table 1:** Characteristics of the included studies

Study	Country	Setting	Study design	Period of EC diagnosis	Patient selection
2004 Koshyama	Japan	Tenri Hospital and Himeji National Hospital	Retrospective cohort	1989–2001	Not specified
2014 Matsuo	USA	Los Angeles County Medical Center	Retrospective cohort	2000–2012	Consecutive
2017 Erkilinc	Turkey	University of Medical Sciences Tepecik Education and Research Hospital	Retrospective cohort	2007–2016	Consecutive
2017 Mao	China	Central Hospital of Lishui City	Retrospective cohort	2006–2013	Consecutive
2017 Zhang	China	Hebei general Hospital	Retrospective cohort	2008–2014	Consecutive
2018 Boonlak	Thailandia	Srinagarind Hospital	Retrospective cohort	2010–2016	Consecutive
2019 Zouzoulas	Greece	“Papageorgiou” Hospital, Thessaloniki	Retrospective cohort	2012–2017	Consecutive
2020 Jonhatty	Australia	Berghofer medical research institute	Retrospective cohort	Not specified	Not specified

**Table 2:** Prevalence of adenomyosis in endometrial cancer patients in the included studies

Study	Total sample	Adenomyosis, *n* (%)	
		Yes	No
2004 Koshiyama	179	29 (16.2)	150 (83.8)
2014 Matsuo	571	271 (47.4)	300 (52.5)
2017 Erkilinc	1134	80 (7.1)	1054 (92.9)
2017 Mao	127	24 (18.9)	103 (81)
2017 Zhang	1584	150 (9.5)	1.434 (90.5)
2018 Boonlak	350	132 (37.7)	218 (62.3)
2019 Zouzoulas	229	64 (27.9)	165 (72)
2020 Jonhatty	1399	572 (40.9)	827 (59)

From studies with extractable data, 64.7% of patients were menopausal, and 19.3% were nulliparous. Of total of EC, 88.1% were endometrioid (88.3% in patients with adenomyosis, and 88% in patients without adenomyosis), 11.3% International Federation of Gynecology and Obstetrics (FIGO) grade 3, 72.1% FIGO stage I (78.5% in patients with adenomyosis, and 68.6% in patients without adenomyosis), 21.6% had myometrial infiltration > 50% and 19.4% lymph vascular space invasion.

**Fig. 2:**
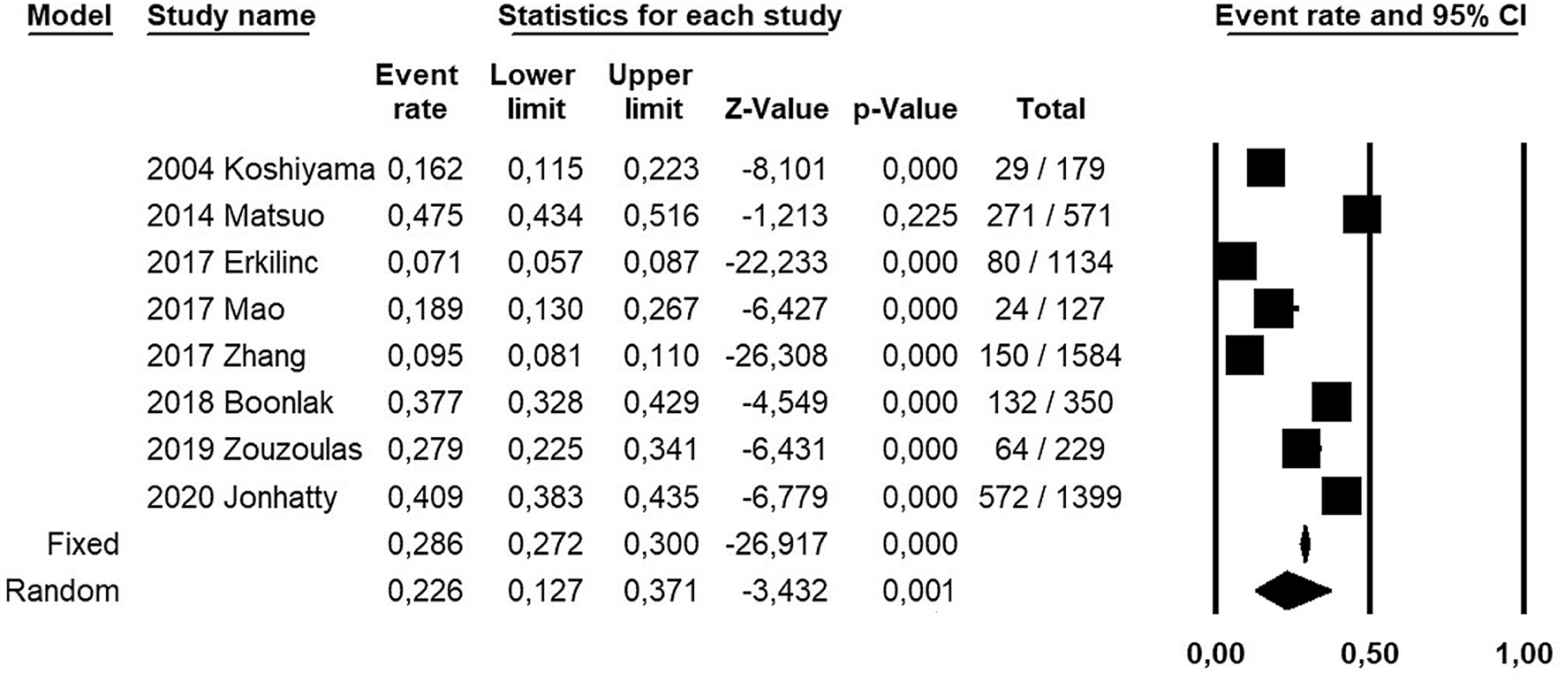
Forest plot of prevalence of adenomyosis in patients with endometrial cancer, for each included study and as pooled estimate

Pooled prevalence of adenomyosis in EC patients was 22.6% (95% CI 12.7–37.1%). Statistical heterogeneity among studies was high (*I*^2^ 99%) (Fig. 2).

**Fig. 3:**
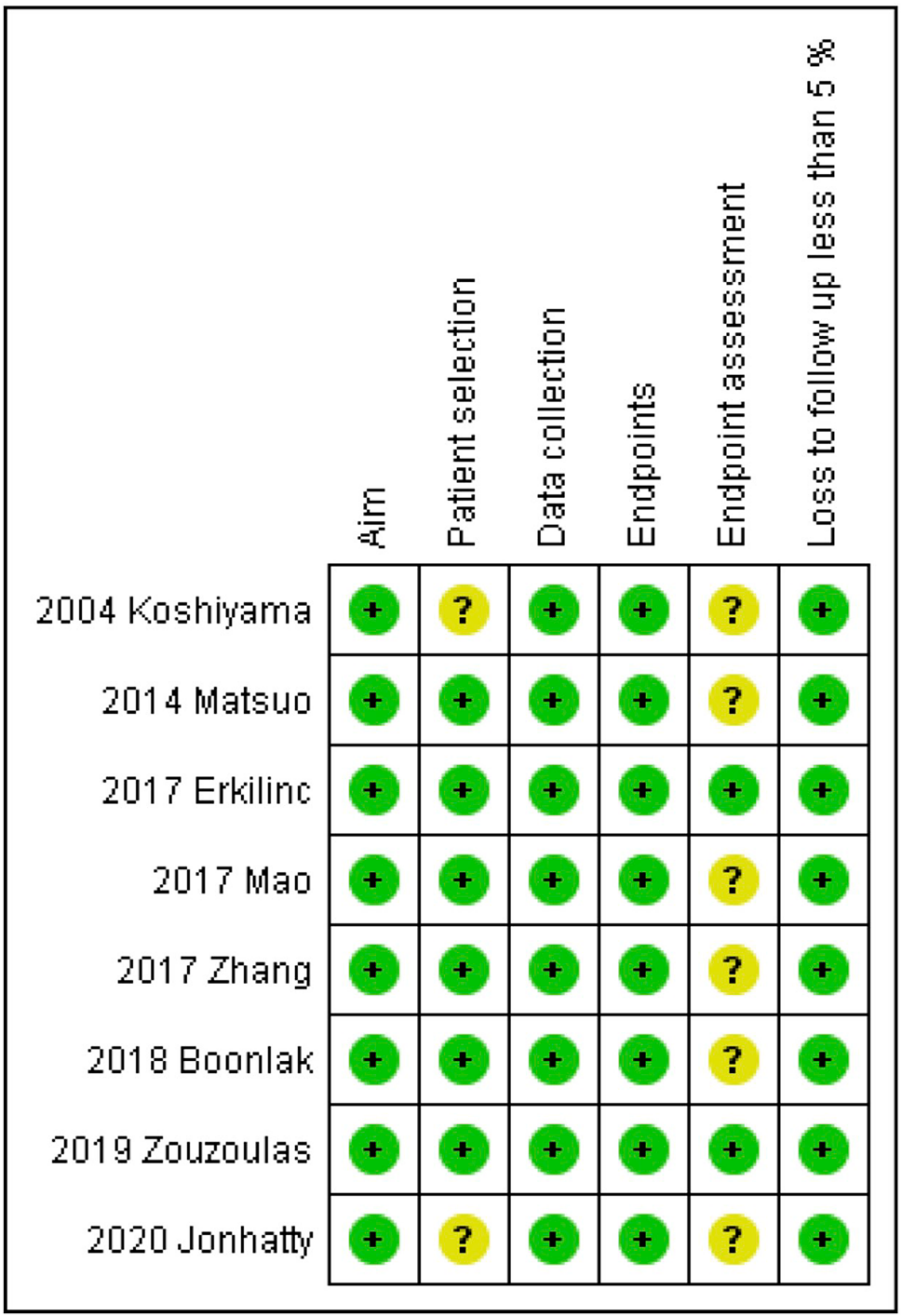
Assessment of risk of bias. Summary of risk of bias for each study; Plus sign: low risk of bias; minus sign: high risk of bias; question mark: unclear risk of bias

**Fig. 4:**
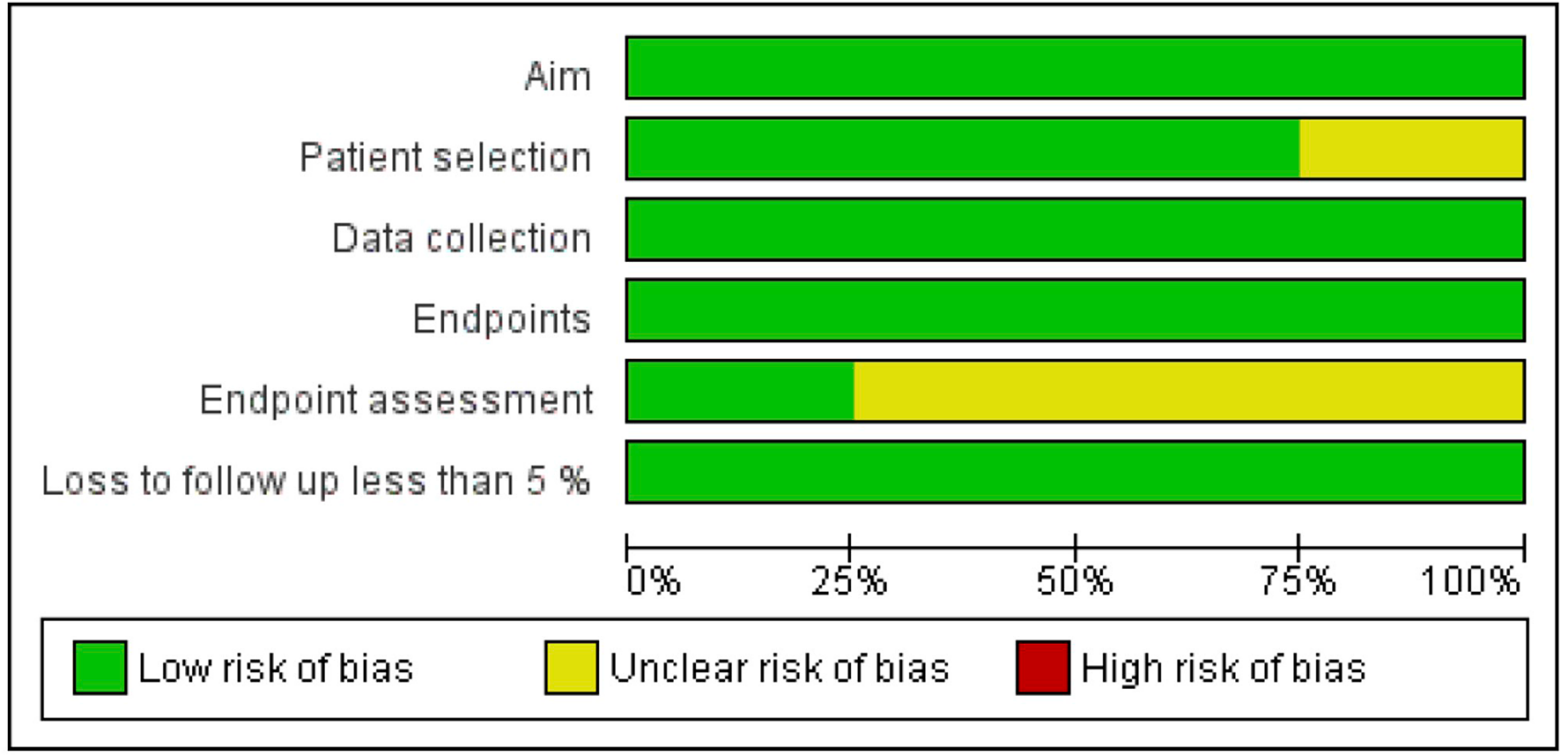
Risk of bias graph about each risk of bias item presented as percentages across all included studies

Regarding the risk of bias within studies assessment, all included studies were judged at “low risk” of bias in the “Aim”, “Data collection”, and “Endpoints” domains. In the “Patient selection” domain, two studies were considered at “unclear risk” of bias because they did not clearly report if all eligible patients were included in the study during the study period [6, 13]. In the “Endpoint assessment” domain, six studies were considered at “unclear risk” of bias because they did not clearly report histological criteria to diagnose adenomyosis [6, 7, 9–11, 13] (Figs. 3, 4).

**Fig. 5:**
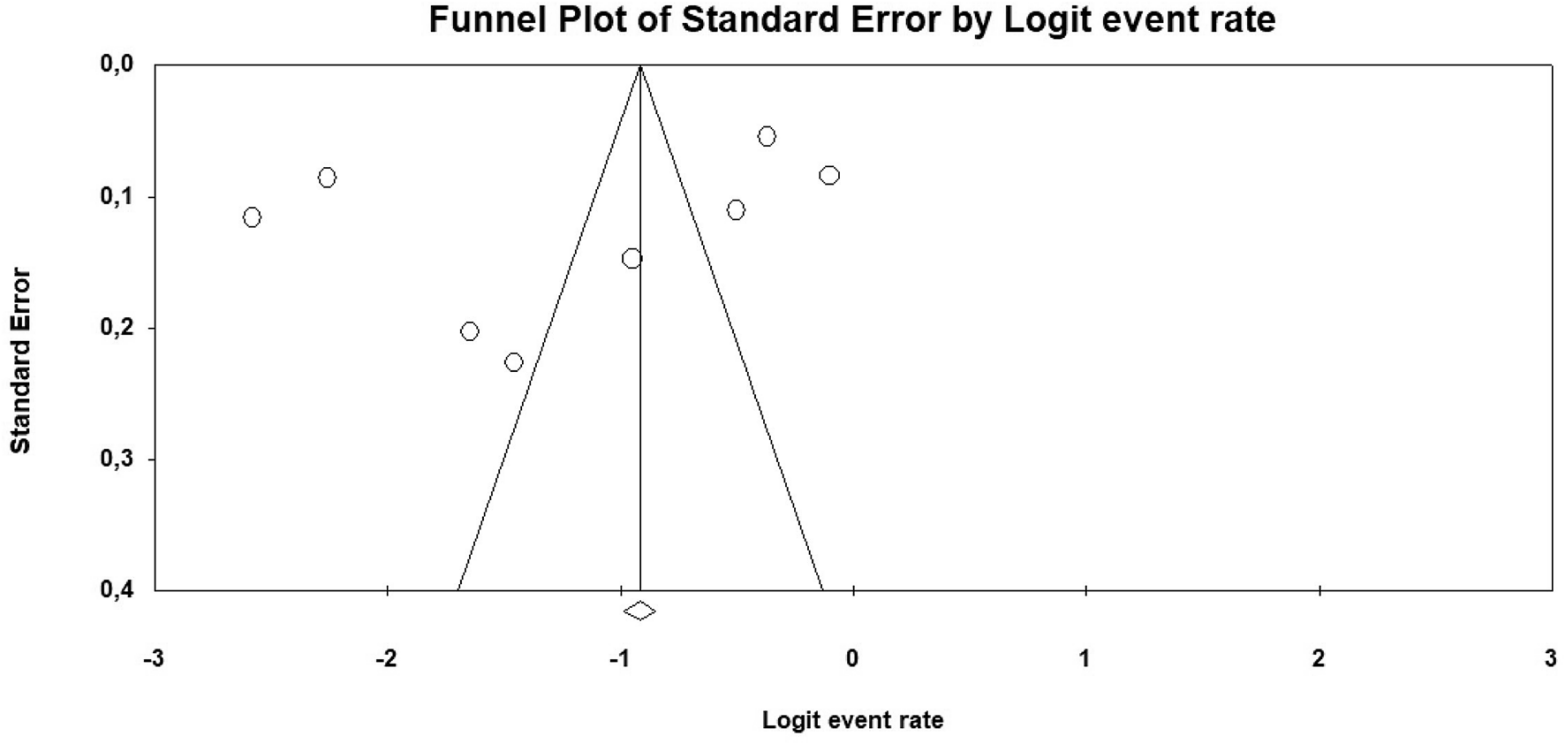
Funnel plot for the assessment of the risk of bias across studies, reporting logit adenomyosis rate on the *x* axis and the standard error on the *y* axis

Regarding the risk of bias across studies assessment, despite outlier studies, the funnel plot indicated that the risk of publication bias was not significant. In fact, it did not show asymmetrical distribution of prevalence values, and the studies with higher standard error did not show higher adenomyosis prevalence (Fig. 5).

## Discussion

### Main findings and interpretation

This study showed that adenomyosis prevalence in EC patients was 22.6%.

Adenomyosis is a common benign gynecologic condition with variable clinical symptoms [15]. However, some studies suggest a potential association with EC [7, 13, 27, 28]. EC is the most common diagnosed gynecological cancer in the developed countries, affecting mostly 50 s and 60 s women [14, 29, 30].

Although the exact pathophysiology underlying the association between the two diseases is unknown, some potential factors have been proposed to explain it. First, the hyper-estrogenic state favors the spread into the myometrium of adenomyosis and, similarly, can promote endometrial cell proliferation and the development of estrogen-related endometrial cancer [2, 14]. Second, adenomyosis is also considered an inflammatory disease secondary to auto-traumatization caused by peristaltic myometrial contraction [15]; likewise, chronic inflammatory condition with secretion of cytokines (Interleukin 6 and 8), chemokines and growth factors (e.g., vascular endothelial growth factor) can facilitate tumor development and dissemination [16, 17]. Third, common mutations in the signaling pathway upregulating cellular proliferation have been found in both the diseases [31]. In particular, the expression of mRNA of Phosphatase and Tensin Homolog (PTEN- mRNA) was observed to be reduced in adenomyosis [32]. Similarly, the gene encoding the PTEN protein has been found mutated in endometrial cancer [33]. Lastly, there is evidence of direct malignant transformation of adenomyosis as Endometrial Cancer Arising In Adenomyosis (ECAIA) [34–36]. This rare entity, accounting for less than 1% of EC and showing poor prognosis, is defined by the following histopathological features: (1) cancer must not be present in the eutopic endometrium or other place in pelvis; (2) cancer must arise from the epithelium of adenomyotic foci and not from another source; (3) endometrial stromal cells have to surround the ectopic endometrial glands to support the diagnosis of adenomyosis [34].

However, in our meta-analysis, prevalence of adenomyosis that we found by pooling data from 8 studies with a total of 5573 EC patients was in line with prevalence reported among hysterectomies for other gynecological conditions (ranged from 21.2% to 36.2%) [37, 38].

Our finding would confirm the hypothesis from Habiba et al. that the co-occurrence of adenomyosis and EC may be due to the high incidence of adenomyosis in peri- and post-menopausal women rather than a direct cause–effect relationship or common pathological pathways among the two diseases [31]. Moreover, the inconsistence of the association between adenomyosis and EC seems to be also supported by a similarity in prevalence of EC histotype and FIGO stages in patients with and without adenomyosis. However, it would be interesting to further investigate the difference in histopathological characteristics in the two groups of patients in future studies, as well as the relationship between the exact localization of the carcinoma and the adenomyosis. In this regard, in a recent meta-analysis, pathogenesis and risk factors of ECAIA were investigated [35]. The malignant transformation of adenomyosis was described as a consequence of endometrial epithelium transition to a monolayer tumor cells [35]. Fibroids, endometrial hyperplasia and polyps seemed to favor the developing of EC within foci of adenomyosis, while endometroid adenocarcinoma, FIGO grade 3 and stage II resulted more common in ECAIA. Moreover, this condition was reported mostly in elderly or post-menopausal patients [35]. Further studies in this field are encouraged.

## Strengths and limitations

To the best of our knowledge, this may be the first systematic review and meta-analysis to provide pooled prevalence of adenomyosis in EC patients. Our results may clarify the association that has been proposed between the two diseases, showing no difference for adenomyosis prevalence between EC and other gynecological conditions requiring hysterectomy. Our findings are supported by an overall good quality of the included studies, as shown in the risk of bias within and across studies assessment. In particular, no study was judged at “high risk” of bias in any domains related to bias, and the funnel plot excluded a significant risk of publication bias.

However, limitations might affect our results. First, histological diagnostic criteria for adenomyosis and tissue sampling methods might affect adenomyosis diagnosis and thus prevalence [15, 39]. In particular, as shown in the risk of bias within studies assessment, some included studies did not report the histological criteria to diagnose adenomyosis [6, 7, 9–11, 13]. This might also explain the high statistical heterogeneity that we found by pooling data. Second, no included study assessed a control group of women without EC, not allowing pooled direct comparisons among adenomyosis prevalence in women with and without EC.

## Conclusion

Adenomyosis prevalence in EC patients was not different from that reported for other gynecological conditions requiring hysterectomy. The supposed association between the two diseases appears unsupported.

## Data Availability

Systematic review and meta-analysis on available studies.
